# The Preservation of the Perineum during the Expulsion of the Fœtal Head

**Published:** 1886-09

**Authors:** 


### THE PRESERVATION OF THE PERINEUM DURING THE EXPULSION OF THE FŒTAL HEAD.

---

TRANSLATED FROM AN ARTICLE BY DOLERIS, IN THE NOUVELLES ARCHIVES D'OBSTETRIQUE ET DE GYNECOLOGIE, BY VIRGIL O. HARDON, M. D., ATLANTA, GA.

---

There exist many methods proposed from time to time for preventing the rupture of the vulvo-vaginal orifice. All play a more or less useful part in the general management, looking toward the protection of the soft parts. But I am satisfied that no one of them by itself is complete or efficacious.

1. There is one which is radically bad, and naturally it is the one most frequently employed, being still recommended and taught. It consists of forcibly applying the palm of the hand at each expulsive effort flat upon the perineum. This is called

"supporting the perineum." That expression should be expunged from obstetrical language, for then perhaps the last remnant of this disastrous method would disappear also. Its most frequent result is that the perineum is torn from top to bottom between the hand which presses and the head which forcibly advances. If this accident does not happen it is because of the extreme suppleness of the tissues, or else because the pressure of the hand upon the head prevents its rapid advance, and so is of benefit in an unexceptional and unintended way.

2. A somewhat better method, taught by some of the pupils of Tarnier after the English accoucheur, Playfair, consists in drawing toward the posterior commissure of the vulva as much as possible of the integument. This is accomplished by placing the palm of the hand upon the perineum, the radial border encircling the lower half of the vulva, the thumb being placed just inside of the tuber ischii of one side and the index and middle fingers just inside that of the other. At each uterine contraction by approximating the thumb and fingers the soft tissues are forcibly drawn toward the fourchette. The object of this procedure is to increase the amount of tissue at the perineum, and in that way to produce a relaxation. It is possible that this method may sometimes be successful in fat women with flabby and elastic skin, but it is a mistake to suppose that success will be the rule. I have often tried this method by itself with the view of determining its value, and I must say that I have no confidence in it. In many cases it is impracticable as well as useless, since the perineal integument is so tense that any folding of the skin is impossible, even in the intervals between the contractions.

3. Finally there is a third method, which consists in dividing the perineum to prevent its being torn. This is like jumping into the water to get out of the rain. It is sometimes spoken of as prophylactic incision or, in more strictly surgical language, episiotomy.

The most frequent laceration of the perineum is at the fourchette. In nine cases out of ten it commences in the mucous membrane of the vagina and extends thence to the skin. It is a laceration by propagation. When it is otherwise the laceration is either unilateral or bilateral and occupies the inner face of the lateral walls of the vagina and of the labia, its direction being parallel or slightly oblique to the axis of the vagina. The maximum of laceration is at the vaginal sphincter. This proves that the resistance is at the vaginal sphincter and that the incision should be made at that point, and not at the free muco-cutaneous border which encircles the head in its passage through the vulva.

What are the evil results of these various lacerations when occurring spontaneously? It is a well-known fact that they differ materially according as the lesion is in the fourchette or in the vaginal walls.

Those of the walls cicatrize very readily, are very easily treated by topical applications, are very little exposed to infection and interfere very slightly with the resistance of the pelvic floor in its *role* of supporting the pelvic organs. Those of the fourchette are often retarded in their cicatrization by the contact of the lochia and their situation at the angle of the vulva. Topical applications are not easily kept in contact with them. They are constantly exposed to the direct absorption of septic products, and in case of non-union and healing by granulation, the vulva is more or less enlarged, and the supporting power of the perineum lessened.

Hence, if prophylactic incision is practiced, it may be done intelligently if not always judiciously. It is the vulvo-vaginal sphincter or the muscular orifice of the vagina which resists, and not the vulvar border. It is therefore necessary that a probe-pointed bistoury should be carried flatwise up to the sphincter in the interval between two contractions. (The sphincter is from three-quarters of an inch to an inch and a quarter from the free border of the vulva.) It is at that point that the incision should be made, and it should embrace the whole thickness of the sphincter. It may thence be carried down to the free border if necessary.

But far more important and effectual are those methods which are applied to the fœtal head, the principal one of which consists in giving the head time enough to make a passage for itself, and in so directing the movement of the fœtus that the maximum of force shall be directed toward the point of greatest resistance, and the weaker parts shall be protected.

To accomplish these purposes, two objects must be kept in view:

1. To modify the force and rapidity of the expulsive contractions of the womb. The object of this is to prevent rupture of the mucous membrane at the vaginal sphincter.

2. To disengage the fœtal head without the aid of the voluntary or involuntary efforts of the parturient woman by an artificial means employed in the interval between two of the final expulsive efforts. The object of this is to prevent rupture of the fourchette—that is to say, the muco-cutaneous angle of the perineum.

The first object, the slow and gradual progression of the head, which is equivalent to the slow and gradual distention of the vulvo-vaginal orifice, is easily accomplished by counter-pressure exerted lightly or forcibly, according to circumstances, by the left hand of the accoucheur upon the occipital extremity of the head during an expulsive pain. During the whole period of expulsion it is necessary to push back the head if it is too rapidly advanced by the uterine contractions. In order that the perineum may remain intact, this contest between the accoucheur and the uterine efforts should last for a half or three-quarters of an hour at least. I always protest against the practice of exhorting the woman to bear down forcibly as soon as the head appears. It is neither necessary nor useful. It is positively injurious since it renders her more liable to a laceration. Its only advantage is to save time for the physician and his assistants.

But when must one cease to push back and restrain the head? I think the pressure should not cease until the time arrives for the employment of the second procedure. I refer to artificial extraction by the hands and fingers, and to rectal expression especially.

We are indebted for the method of rectal expression to Olshausen and Ahlfeld. It consists in introducing two fingers into the rectum of the woman toward the end of a pain and carrying them up to the mouth or under the chin of the child. By welldirected pressure downward and forward, the extension of the head may be accomplished so that it will escape from under the pubic arch. This manœuvre is very easy of accomplishment, for the anus is widely dilated and is free from fæcal matter. It is not painful. I have never heard women complain much during the process. Care must be taken that the extension should be slowly accomplished; otherwise an injury to the fourchette or the lateral walls of the vagina may result. For this reason it is necessary to combine traction and pressure by means of the fingers in the rectum.

In regard to the time for the employment of this procedure, two points are to be borne in mind:

1. It must be undertaken only in the interval between two contractions. The pain must be on the wane or entirely ceased. For the greater security it is best to direct the woman to abstain from bearing down. Up to this time voluntary efforts have been necessary. The accoucheur has regulated and encouraged them. But at this point they become useless and even dangerous.

2. In order to judge when the propitious moment has arrived, it is necessary to wait for certain conditions to be fulfilled. After a careful study of the subject in primiparæ, I am satisfied that when the posterior angle of the anterior fontanelle is plainly visible at the commissure, it is time to employ rectal expression and to accomplish artificial delivery by the combined use of two hands. At that moment the largest circumference of the head is engaged or about to be engaged; it may have even passed the point of resistance; this depends upon the conformation of the head.

Thus performed, rectal expression, which is my habitual practice in primiparæ with small vulvæ, has given excellent results in my hands. No other argument is needed to recommend it. If figures were necessary, I could furnish comparative statistics of the three following methods: Spontaneous disengagement, various methods of supporting the perineum and rectal expression, to prove that the last method is the one in which there is least liability to rupture of the perineum.

I conclude then that the best method of protecting the perineum is the following:

1. To prevent a deep rupture from commencing at the vaginal sphincter during the too rapid progress of the head at that point, regulate and control the passage of the head through this part of the canal, which in primiparæ should occupy from one-half to three-quarters of an hour. The distention of the parts should be gradual, and the tearing of the mucous membrane should be avoided. To secure these objects, control the escape of the head by pressing it back—not in a hap-hazard way, but at each new uterine contraction.

2. To prevent a rupture of the fourchette from occurring alone, or as the result of a slight lesion of the mucous membrane in a part of the vagina concealed from the eye of the physician, wait until the largest circumference of the head is engaged, and when a half of the anterior fontanelle has emerged, execute the manœuvre of rectal expression.

3. If, through fear or through haste, a liberative incision is decided upon, wait as long as possible, and then make it laterally and through the vaginal sphincter by means of a probe-pointed bistoury introduced flatwise, at the end of a contraction, while the part is still tense.

